# Increase in NDM-1 and NDM-1/OXA-48-producing *Klebsiella pneumoniae* in Germany associated with the war in Ukraine, 2022

**DOI:** 10.2807/1560-7917.ES.2022.27.50.2200926

**Published:** 2022-12-15

**Authors:** Mirco Sandfort, Jörg B Hans, Martin A Fischer, Felix Reichert, Martina Cremanns, Jessica Eisfeld, Yvonne Pfeifer, Annika Heck, Tim Eckmanns, Guido Werner, Sören Gatermann, Sebastian Haller, Niels Pfennigwerth

**Affiliations:** 1 Department of Infectious Disease Epidemiology, Robert Koch Institute, Berlin, Germany; 2 German National Reference Centre for Multidrug-resistant Gram-negative Bacteria, Department of Medical Microbiology, Ruhr-University Bochum, Bochum, Germany; 3 Department of Infectious Diseases, Robert Koch Institute, Wernigerode, Germany

**Keywords:** Klebsiella pneumoniae, NDM-1, NDM-1/OXA-48, Ukraine, Germany, carbapenemase-producing Enterobacterales, genomic surveillance, antimicrobial resistance

## Abstract

In 2022, German surveillance systems observed rapidly increasing numbers of NDM-1- and NDM-1/OXA-48-producing *Klebsiella pneumoniae*, which may in part reflect recurring pre-pandemic trends. Among these cases, however, a presence in Ukraine before diagnosis was frequently reported. Whole genome sequencing of 200 isolates showed a high prevalence of sequence types ST147, ST307, ST395 and ST23, including clusters corresponding to clonal dissemination and suggesting onward transmission in Germany. Screening and isolation of patients from Ukraine may help avoid onward transmission.

Surveillance systems in Germany revealed an increase in NDM-1 and NDM-1/OXA-48-producing *Klebsiella pneumoniae* since March 2022. It coincided with the war in Ukraine and the arrival of refugees and evacuated patients to Germany. In a nationwide investigation, we combined epidemiological and genomic analyses to delineate transmission patterns and infer clinical care recommendations.

## Epidemiology of carbapenemase-producing Enterobacterales in Germany

**Figure 1:**
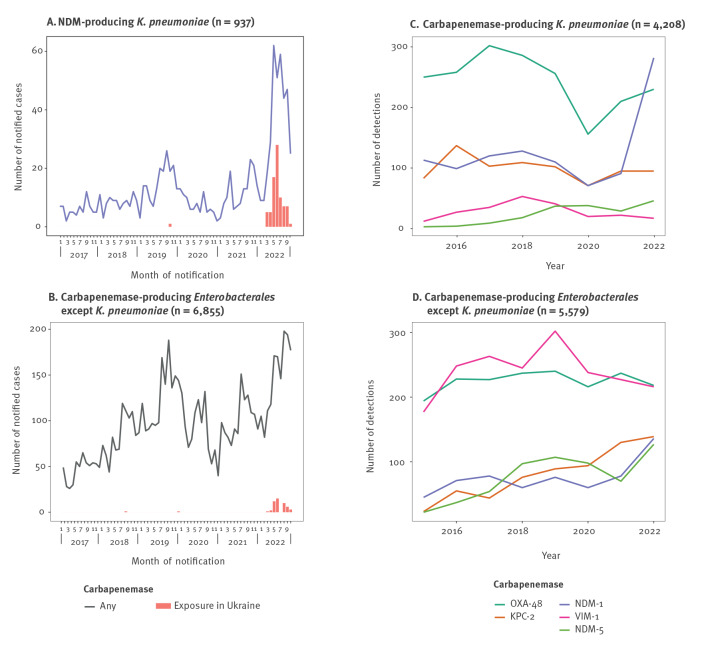
Notifications of NDM-producing *Klebsiella pneumoniae* and other carbapenemase-producing Enterobacterales and carbapenemase detections for multidrug-resistant Gram-negative bacteria, Germany, 2022

Since 2016, infections or colonisations with carbapenemase-producing Enterobacterales (CPE) have been notifiable in Germany. After a period of reduced incidence since the beginning of the coronavirus disease 2019 (COVID-19) pandemic [1,2], notifications of NDM-producing *K. pneumoniae* have exceeded pre-pandemic trends since March 2022, in contrast to CPE excluding *K. pneumoniae* (Figure 1 panels A and B). In the Supplement, part S1, we provide additional detail on the differing trends and corresponding trend analyses.

Panels A and B: data from the national notifiable diseases surveillance system. Panels C and D: detections at the National Reference Centre.

Particularly for case notifications of NDM-producing *K. pneumoniae* (Figure 1A), presence in Ukraine before diagnosis was common: 80 of 335 (24%) notifications since calendar week 10/2022, compared with one of 584 (0.2%) between 2017 and 2021. For CPE excluding *K. pneumoniae* (Figure 1B), respectively 68 of 2,306 (3%) and two of 12,045 (< 0.1%) cases were from Ukraine in these two time periods, (chi-squared test for exposure in Ukraine in 2022 among notifications of NDM-producing *K. pneumoniae* vs other CPE: p < 0.001). Notifications do not always specify a recorded presence abroad as provenance, hospitalisation or travel. However, it is likely that notified cases since March 2022 with a recorded presence in Ukraine represent predominantly refugees or evacuated patients.

At the same time, the German National Reference Centre (NRC) for multidrug-resistant Gram-negative bacteria detected NDM-1-positive *K. pneumoniae* isolates in unprecedented numbers compared with other carbapenemases and other years (Figure 1C) [3]. NDM-1-positive isolates of other Enterobacterales increased as well, but to a lesser extent (Figure 1D).

 For 75 of 335 (22%) NDM-producing *K. pneumoniae* notifications since week 10/2022, the carbapenemase was specified as NDM-1. Of the remaining notifications, 250 (75%) were unspecified, eight had NDM-5, one NDM-4 and one NDM-9. Prior presence in Ukraine was reported for 24 of the 75 (32%) NDM-1 notifications (Table). Compared with other exposure locations, cases from Ukraine were younger (p < 0.01) and tended to be more often colonised, detected via screening (p = 0.24). Of all infections, wound infections predominated, in contrast to urinary tract infections among cases with exposure locations elsewhere, suggesting a considerable part of notifications related to evacuated patients [4,5]. Co-production of an OXA-48(-like) carbapenemase was found in 12 of 24 cases from Ukraine. Cases were notified from 11 of 16 German federal states and throughout the weeks 10–43 in 2022.

**Table:** Demographic and clinical data and sample characteristics for notified *Klebsiella pneumoniae* with a confirmed NDM-1 carbapenemase, Germany, weeks 10–43/2022 (n = 75)

Patient information	Total		With reported country location of exposure		
	n	%	Germany or foreign country other than Ukraine	Ukraine	
				All	Co-production of NDM-1 and OXA-48(-like) carbapenemases
Sex	75	100	19	24	12
Female	32	43	11	8	4
Male	42	56	8	15	7
Missing	1	1	0	1	1
Median age in years (IQR)	49 (34–66)		63 (46–72)	41 (34–47)	40 (29–45)
Missing	0		0	0	0
Sampling material ^a^					
Blood/liquor	7	9	2	1	1
Urine	14	19	9	1	0
Wound	17	23	1	9	4
Screening/stool	31	41	6	11	5
Other or missing	6	8	1	2	2
Infections vs colonisations^b^					
Infections	39	52	13	11	5
Colonisations	34	45	6	13	7
Missing	2	3	0	0	0

IQR: interquartile range.

^a^ Sampling material was documented as multiple choice but evaluated here by selecting the first material mentioned in the order: blood/liquor, urine, wound, screening/stool.

^b^ Infection vs colonisation was inferred from the sampling material (from blood/liquor/urine/wound vs screening/stool, respectively), and from the classification as in the notification if the information on material was missing.

## Whole genome sequencing and cluster detection

Between January and September 2022, the NRC received 330 non-duplicate NDM-1- and NDM-1/OXA-48-producing *K. pneumoniae* isolates. Of these, 200 isolates, comprising 66 NDM-1/OXA-48-producing and 134 NDM-1-producing *K. pneumoniae*, were subjected to Illumina (Illumina, San Diego, United States) whole genome sequencing. Sample selection prioritised NDM-1/OXA-48-producing isolates and NDM-1-producing isolates from cases from Ukraine and known hospital origin. The remaining isolates were selected randomly.

**Figure 2:**
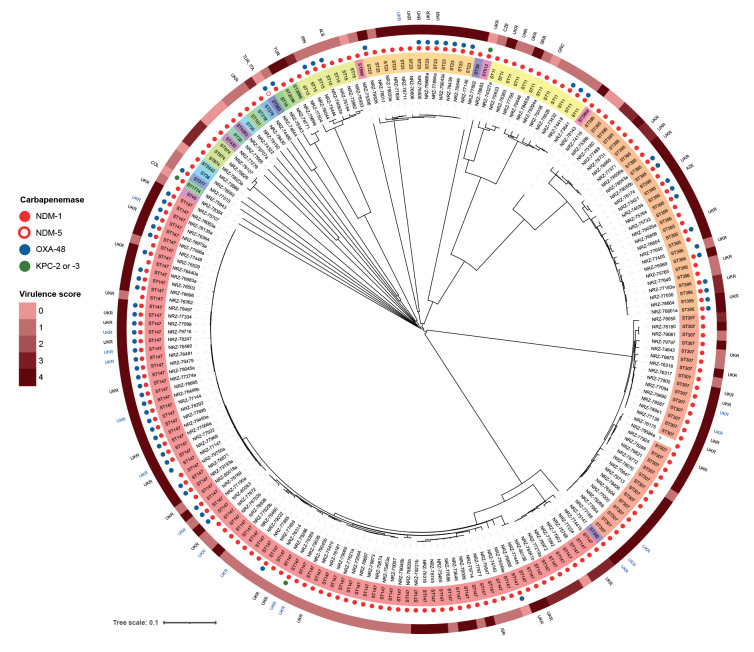
Phylogenetic tree of NDM-1 and NDM-1/OXA-48-producing *Klebsiella pneumoniae* isolates, Germany, January–September 2022 (n = 200)

Analyses of de novo assemblies included multilocus sequence typing (MLST) and core genome (cg)MLST using the *K. pneumoniae* sensu lato scheme (2,358 loci) as implemented in the SeqSphere+ software version 8.4.1 (Ridom, Muenster, Germany). Among the 200 isolates, we identified 27 sequence types (ST) with a predominance of ST147 (88/200; 44%), ST307 (28/200; 14%) and ST395 (27/200; 14%) (Figure 2). Thirteen (7%) isolates belonged to ST23, often associated with hypervirulence [6,7].

We determined pairwise allelic differences between isolates and calculated a neighbour-joining tree using SeqSphere+. Metadata were annotated using Interactive Tree OF Life [20]. Sequence types (ST) are colour-coded. Coloured circles: detected carbapenemases; reddish band: Kleborate-derived virulence score [8]; 3-letter country code: presence abroad before diagnosis, as documented for isolates at the NRC (black) or matched notifications (blue).

OXA-48 was primarily identified among ST147 isolates, but also in other ST. In silico analyses revealed the presence of one or more additional extended-spectrum beta-lactamase (ESBL) genes (*bla*_CTX-M_, *bla*_SHV_, *bla*_TEM_) in almost all isolates (199/200). The detailed results of detected ESBL genes are appended in the Supplement, part S2. Kleborate [8] identified the presence of genes for the siderophores aerobactin and yersiniabactin, resulting in a virulence score of up to 4 throughout the phylogenetic tree. Colibactin-encoding genes were not found, hence, no virulence scoring of 5 was assigned.

Prior presence in Ukraine was known from case information at the NRC or from the notifications matched to isolates for 60 of 200 (30%) isolates and across all major ST: for 29 of 88 ST147, for nine of 27 ST395, for five of 13 ST23 and for 11 of 28 ST307. Overall, the diversity of ST indicates that the observed increase was not due to a single transmission cluster but rather suggests a high incidence of NDM-1 and NDM-1/OXA-48-producing *K. pneumoniae* in Ukraine.

**Figure 3:**
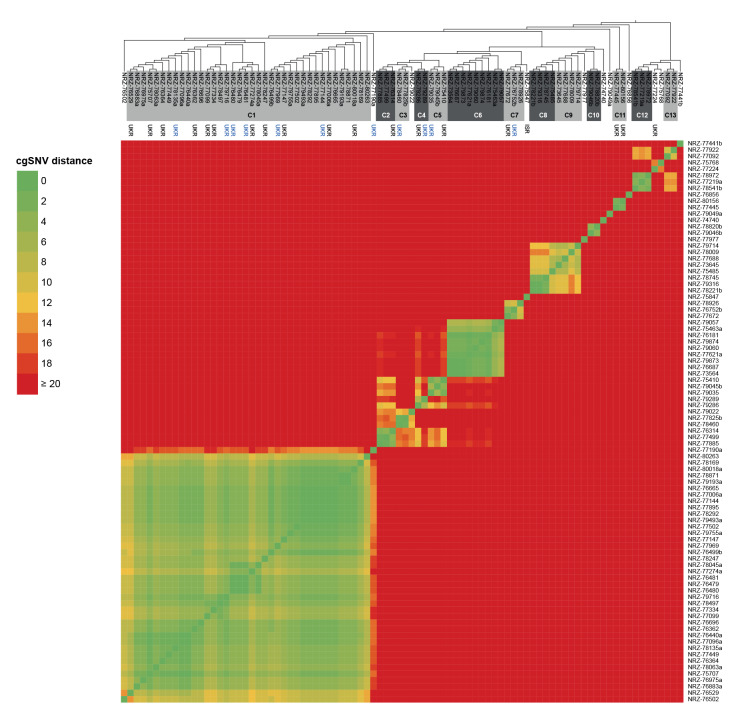
Phylogenetic tree and heatmap based on pairwise single nucleotide variant (cgSNV) differences between isolates of *Klebsiella pneumoniae* ST147, Germany, January–September 2022 (n = 88)

Results of cgMLST also revealed clusters of close genetic relatedness for many of the different ST, particularly among ST147. To further examine genetic relatedness, we performed single nucleotide variant (SNV)-based analyses within genes of the cgMLST scheme, as implemented in SeqSphere+. Because there is no established threshold for clonal relatedness, we performed molecular typing by *Xba*I-macrorestriction and pulsed-field gel electrophoresis on 14 NDM-1/OXA-48-producing *K. pneumoniae* ST147 isolates clustering within the phylogenetic tree of the cgMLST analysis. Following defined criteria [9], these isolates were clonally related as revealed by identical banding patterns (data not shown). In the SNV-based analysis, these 14 isolates showed a maximum of 12 cgSNV, which we therefore set as threshold to define clonality. By applying this upper limit, we identified clusters suggestive of clonal dissemination (Figure 3).

Isolates of clusters differing by a maximum of 12 cgSNV in grey (see text for details). 3-letter country code: presence abroad before diagnosis, as documented for isolates at the national reference centre (black) or matched notifications (blue).

The largest cluster C1 included 38 isolates, sampled from April to September 2022. Hospital locations in Germany and from where cases were notified scattered nationwide. Prior presence in Ukraine was reported for 17 of the 38 cases, suggesting a cluster of refugees from Ukraine or evacuated patients and reflecting transmission in Ukraine. For two of the 38 cases, with sampling dates in May and September, however, reported exposure locations were exclusively in Germany, indicating potential onward transmission. In contrast, cluster C6 included cases exclusively from two neighbouring hospitals in Germany without reported links to Ukraine, potentially representing transmission within Germany. Further investigations of these clusters have begun to elucidate chains of transmission.

## Antimicrobial resistance

In phenotypical tests, nine isolates representative of ST147, ST395, ST307 and ST23 were resistant to meropenem and imipenem, piperacillin/tazobactam, ceftazidime, aztreonam, and to the inhibitor combinations ceftazidime/avibactam, meropenem/vaborbactam and imipenem/relebactam (testing and interpretations was done according to the European Committee on Antimicrobial Susceptibility Testing (EUCAST) v12.0 [10]). They were also resistant to fluoroquinolones and cefiderocol (or within the area of technical uncertainty), which in case of cefiderocol has been previously reported for several NDM producers [11,12]. For many isolates, we detected low minimum inhibitory concentration (MIC) of colistin (MIC ≤ 2 mg/L) and tigecycline (MIC ≤ 0.5 mg/L); however, neither of these drugs is recommended in serious infections [13]. Checkerboard titrations with aztreonam (0.5–32 mg/L) and ceftazidime/avibactam (0.25–64 mg/L and 4 mg/L, respectively) were done and the addition of ceftazidime/avibactam to aztreonam resulted in markedly reduced MIC to aztreonam (MIC of aztreonam in combination ≤ 0.5 mg/L), suggesting synergism and a reduction into the therapeutically usable range as reported previously [14,15].

## Discussion

Since March 2022, NDM-1-producing *K. pneumoniae* increased rapidly in Germany and in these cases, presence in Ukraine before diagnosis was frequently reported. Ukraine reported high AMR proportions in Gram-negative bacterial isolates previously (mostly bloodstream infections) to the Central Asian and European Surveillance of Antimicrobial Resistance (CAESAR) network, including *K. pneumoniae* with resistance to carbapenems in 64% and *Acinetobacter* spp. with resistance to carbapenems in 73% in 2021 [16]. As a result, the European Centre for Disease Prevention and Control (ECDC) recommended in March 2022 to screen for carriage of multidrug-resistant organisms (MDRO) in patients transferred from hospitals in Ukraine or with a history of hospital admission in Ukraine and to implement multimodal infection prevention and control (IPC) measures [17]. The IPC guidelines in Germany generally recommend screening for MDRO in individuals with reported hospitalisation in countries with high antimicrobial resistance rates, including Ukraine [18,19]. However, the increasing incidence and proportion of NDM-producing *K. pneumoniae* in cases from Ukraine have not occurred for other CPE, suggesting that the increase does not only stem from intensified screening of patients from Ukraine. Diagnostic laboratories in Germany should voluntarily send carbapenem-resistant isolates to the NRC for carbapenemase specification and notify all cases to the mandatory notification system. But there is a known under-ascertainment in both data sources and so far, isolates and notifications cannot be linked by a common identifier. Linking the information of the two used data sources for cluster analyses as we present here is challenged by the lack of a unique common identifier. We used several variables for matching, which is less reliable.

Whole genome sequencing revealed the predominance of *K. pneumoniae* sequence types ST147 and ST307, both of which are known to be successful epidemic clones. Although detailed genomic analyses indicate the clonal relatedness of NDM-1 and NDM-1/OXA-48-producing *K. pneumoniae* ST147 isolates, a comparison with international ST147 isolates will evaluate whether the close genetic relatedness reflects transmissions of strains or simply is a characteristic of the epidemic clone. 

## Conclusions

NDM-1-producing *K. pneumoniae* has become more frequent in Germany, particularly in 2022. Our findings suggest an elevated proportion of NDM-1 and NDM-1/OXA-48-producing *K. pneumoniae* among refugees or evacuated patients from Ukraine, adding to the baseline incidence in Germany. We need to further investigate if high transmission occurs in single or multiple treatment centres in Germany or Ukraine to specifically assess how best to support IPC and outbreak control. Adherence to recommended IPC measures in Germany and other European countries needs to be stressed to prevent onward transmission. The combination of aztreonam with ceftazidime-avibactam may be considered for the treatment of severe infections caused by these *K. pneumoniae* strains.

## Supplementary Data

243000 Supplementary Material 1
