# Supplementary material for: Increase in NDM-1 and NDM-1/OXA-48-producing Klebsiella pneumoniae in Germany associated with the war in Ukraine, 2022
Source: Euro Surveill. 2022 Dec 15;27(50):2200926. doi: 10.2807/1560-7917.ES.2022.27.50.2200926 (PMC9808319; doi:10.2807/1560-7917.ES.2022.27.50.2200926)
Supplement: Supplementary Material 1 [file 22-00926_SANDFORT_Supplement.pdf]

Disclaimer: This supplementary material is hosted by *Eurosurveillance* as supporting information alongside the article “Increase in NDM-1 and NDM-1/OXA-48-producing *Klebsiella pneumoniae* in Germany associated with the war in Ukraine, 2022”, on behalf of the authors, who remain responsible for the accuracy and appropriateness of the content. The same standards for ethics, copyright, attributions and permissions as for the article apply. Supplements are not edited by *Eurosurveillance* and the journal is not responsible for the maintenance of any links or email addresses provided therein.

S1 Trend analyses of monthly notifications of NDM-producing *K. pneumoniae* and carbapenemase-producing Enterobacterales excluding *K. pneumoniae*

The average of 44 notifications per month in March-September 2022 was significantly elevated from the expected average of 28 [95%-prediction interval 18-38], based on a linear trend fitted to notifications pre-COVID-19 (2017–2020). In contrast, the average of 158 notifications per month for CPE excluding *K. pneumoniae* (Figure 1B) was lower than the expectation of 246 [196-296].

S2 Phylogenetic tree and results of *in silico* analyses of beta-lactamase genes in NDM-1 and NDM-1/OXA-48-producing *Klebsiella pneumoniae* isolates from Germany, 2022 (n = 200)

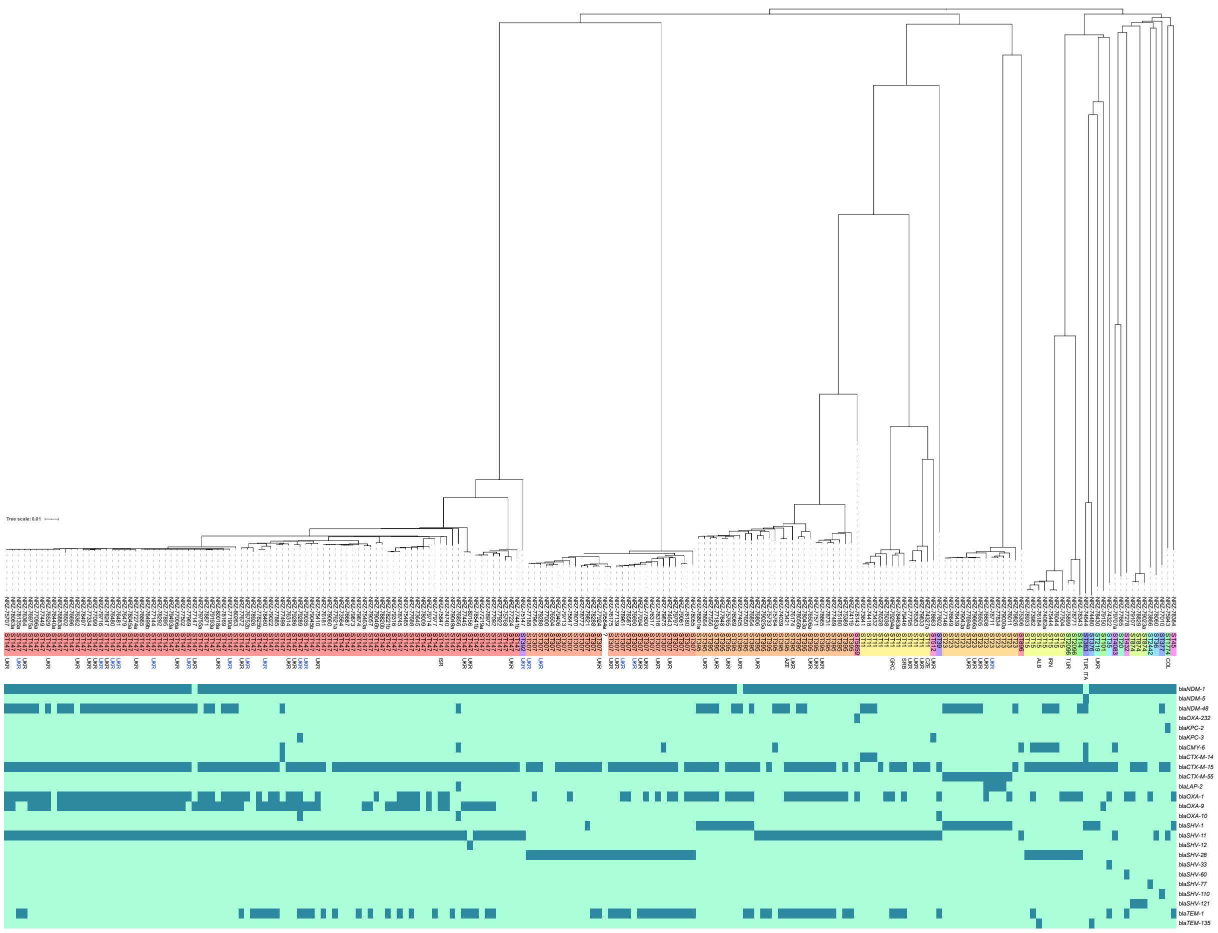

SeqSphere+ software version 8.4.1 was used to determine pairwise allelic differences between isolates and calculate a neighbour-joining tree. Metadata were annotated using Interactive Tree OF Life (1). Sequence types (ST) are colour-coded and exposure abroad is given by the 3-letter country code, as documented on isolates sent to the reference centre (black font) or notifications matched to the isolates (blue). Gene matrix shows the presence (dark green) or the absence (light green) of beta-lactamase genes, as identified by NCBI AMRFinderPlus integrated in the SeqSphere+ software version 8.4.1.

References

(1) Ivica Letunic, Peer Bork, Interactive Tree Of Life (iTOL) v5: an online tool for phylogenetic tree display and annotation, Nucleic Acids Research, Volume 49, Issue W1, 2 July 2021, Pages W293–W296, https://doi.org/10.1093/nar/gkab301
